# Socioeconomic Barriers to Rhegmatogenous Detachment Surgery in Brazil

**DOI:** 10.1155/2014/452152

**Published:** 2014-11-23

**Authors:** Pedro Carlos Carricondo, Tatiana Tanaka, Suellen Tiemi Shibata, Leandro Cabral Zacharias, Thiago Aragão Leite, Maria Fernanda Abalem, Walter Y. Takahashi

**Affiliations:** ^1^Ophthalmology Service of the Clinical Hospital, University of São Paulo, São Paulo, SP, Brazil; ^2^Retina and Vitreous Department, Ophthalmology Service of the Clinical Hospital, University of São Paulo, São Paulo, SP, Brazil; ^3^University of São Paulo Medical School, Rua Capote Valente 171, Apartment 62, 05409-000 São Paulo, SP, Brazil

## Abstract

*Purpose*. To verify access barriers patients with retinal detachment face to arrive at a reference center and to evaluate patients' knowledge about the disease. *Methods*. Transversal study that applied a questioner to 65 patients of the Clinical Hospital of the University of Sao Paulo with retinal detachment between February and August of 2010. *Results*. Reasons for not performing the surgery in other services were as follows: 47% were referred because there was not vitreoretinal surgeon at original service; 27% could not afford the surgery, had no health insurance, or had no coverage at health insurance plan for the procedure. Time between the first symptom and the arrival at our service was as follows: 18 patients arrived in up to 7 days; 35 between 8 and 30 days; 8 between 31 and 90 days; 5 in more than 90 days. Reasons for delay were as follows: 70% did not know how serious the pathology was; 56% thought that it had spontaneous cure; 16% did not have money to pay for ophthalmic evaluation, 10% did not know where to go and 24% for other reasons. *Conclusion*. Educational programs about disease and measures to optimize the referral to specialized services are needed to accelerate the treatment of patients with rhegmatogenous retinal detachment.

## 1. Introduction

Rhegmatogenous retinal detachment (RD) is one of the leading causes of vision loss in all countries around the world [1]. Prompt diagnosis and treatment have a significant effect on the outcome.

Since 1970, epidemiologic studies have been conducted in ophthalmology to examine patient characteristics, risk factors, and prognosis. However, there are few studies focusing on the referral routes of patients with retinal detachment from the diagnosis to surgery, trying to determine the length and source of delays between the onset of symptoms and the arrival to the surgical unit. In Brazil, there are no national statistics analyzing this disorder [2]. Identifying the barriers and the needs of a population is essential to plan the national health system actions [3, 4].

The purpose of this study is to determine the reasons for delay in precocious treatment and to evaluate the perceptions and knowledge of patients with RD about their pathology at the admission in a tertiary care ophthalmic unit in Brazil.

## 2. Methods

This study was approved by the Ethic Committee of the Hospital das Clínicas da Faculdade de Medicina da Universidade de São Paulo (HC-FMUSP), where it was conducted.

A structured questionnaire was applied to patients admitted to the ward of the HC-FMUSP with the diagnosis of RD over a prospective period from February to August 2010. The interviews were carried out by two of the authors (TT, STS), focusing on the patient's symptoms, their perceptions of the severity of the disease, the pathway from the diagnosis to the arrival at HC-FMUSP, and the time from the onset of the symptoms to presentation at our facilities.

The inclusion criteria were as follows:

1. minimum age of 18 years or the authorization of the legal guardian for study participation;
2. rhegmatogenous retinal detachment with surgical repair indicated by a staff doctor of the Retina and Vitreous Department of the HC-FMUSP;
3. signature of the free informed consent term;
4. capacity to understand the questions asked.

The exclusion criteria were as follows: previous rhegmatogenous retinal detachment surgery; difficulty in the comprehension of the questions during the interview; absence of a legal guardian to answer the questionnaire when the patient was not able to answer it alone.

## 3. Results

Sixty-five patients accomplish the inclusion criteria and were included in this study. Forty-four patients (67,7%) were men and 21 (32,3%) women, with a mean age of 49,97 ± 14,45 years.

The first perceived symptoms were loss of visual acuity (67%), visual field scotoma (40%), floaters (37%), photopsias (29%), and symptoms not related to the retinal detachment (9%), such as pain and ocular discomfort. Only 15% of the interviewed patients had floaters and/or flashes without visual acuity loss and/or scotomas.

Just 15% of the patients came directly to the Hospital das Clínicas; the other 85% were first seen by other ophthalmological services. The mean time from the beginning of the symptoms to the first complete ophthalmological evaluation for the patients that first came to the Hospital das Clínicas was 65,00 ± 89,28 days, and for the patients referred from other services it was 27,07 ± 146,36 days. The mean time from diagnosis in other services to examination at Hospital das Clínicas was 23,45 ± 54,08 days. 64,6% of the patients sought assistance in the first week of symptoms, but only 28,6% of them were examined at the Hospital das Clínicas within a week.

**Figure 1:**
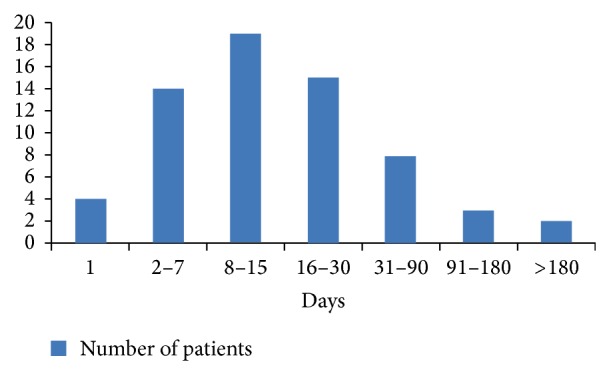
Time, in days, from the beginning of the symptoms to the arrival at the Hospital das Clínicas.

Figure 1 shows the time interval between the first symptoms and the patient arrival at the Hospital das Clínicas.

The reasons patients did not have their surgery performed at the local of first diagnosis (in case of referred patients) were as follows: no retinal specialist was at the site (47%); they could not afford the surgery in a private practice facility (24%), or the private health insurance did not cover for the surgery (29%).

The causes of the delay in medical care to patients that had more than 1 week of interval between the symptoms and the diagnosis are seem in Table 1. Main causes are: did not think it was a really severe problem (70%), thought it would heal by itself (56%), did not have money to go to the doctor office (16%), did not know where to go (10%), and other causes (24%), such as lack of eye doctors at the city of origin.

## 4. Discussion

The increasing demand for ophthalmic care results in delay for patient diagnosis and treatment at the public health system of Brazil [2], as well as in other developing countries [1]. Retinal detachment is a sight threatening condition, and the visual prognosis depends directly on the timing for correct diagnosis and surgical treatment. Moreover, the barriers to vitreoretinal evaluation and surgical intervention must be well known when planning requirements and resources [2].

RD surgery requires tertiary hospital facilities and specialized medical care, which is rare in developing countries, what makes it hard to achieve an appointment because there are few centers with trained retinal surgeons and vitreoretinal surgical equipment available [1]. In Brazil, the scenario is not different, in part because of the dimensions of the country and the large population.

A previous study in another region of Brazil with similar socioeconomic profile showed an incidence of 9,2 new cases of RD with surgical indication per 100.000 habitants/year [2]. Even if we do not consider patients coming from other states of Brazil, as many as 2.300 surgical appointments per year to take care of the new RD cases just in the São Paulo metropolitan area were expected.

This large burden increases the importance of prompt perception of initial RD symptoms by patients and where to find prompt care, in order to achieve better visual outcomes with the surgical treatment [5]. The final visual results are directly dependent on the timing of the surgical repair, especially when the macula is attached. Macular detachment is a key indicator of visual prognosis, since once the macula detaches, less than 75% of patients will achieve a visual acuity of 6/12 or better [5].

Unfortunately, the most common symptom perceived by the patients in this study was visual acuity loss, which corresponds to an advanced course of the disease with a probable macular detachment. This data is similar to what is found even in developed countries [5]. Educational campaigns and periodic examination of patients at risk could be effective ways to address those issues [4–6].

The premonitory symptoms, despite not always present, should be emphasized in public health system in order to provide an opportunity to educate patients [5]. The delay in presentation of RD cases is not apparently related to socioeconomic status, but to the low awareness of population about presenting symptoms [4, 5].

Most of the patients attended other centers of low complexity, as proposed by the Brazilian public health system, but the patients from our study took in average almost one month to be examined in a tertiary center. Possible explanations for this delay from the first diagnose to an appointment at a tertiary center could be misdiagnose of the disease; lack of information about the availability of treatment; or the mechanism of accessing the tertiary service by the general ophthalmologist [6].

Another important cause of delay in presentation was the lack of information from the patients about the severity of the symptoms. Most patients thought that a RD would not be a real problem and that it would heal by itself. It is interesting that the data are very similar to series in developed countries [4, 6]. Other causes of delays, such as difficulty in transportation and need of a care person, are also similar to what is found in other European or American series [6].

## 5. Conclusion

The prognosis of retinal detachment is related to prompt repair, especially if the macula is attached. Measures must be taken to eliminate avoidable delays. Patient and medical education about the disease symptoms and severities could potentially reduce the time between the first symptoms and specialized retinal consult. The public health system must be structured, with the accessing ways known by the attending doctors in every level of health care complexity systems.

## Figures and Tables

**Table 1:** Mentioned reasons for delay in seeing a doctor after starting symptoms.

Reasons for delay in seeing a doctor	
Do not know where to go	10%
Thought that it was not a severe condition	70%
Thought that it would heal itself	56%
Do not have money to go to doctor	16%
Other reasons (do not have doctor in living city)	24%

## References

[1] Yorston D., Jalali S. (2002). Retinal detachment in developing countries. *Eye*.

[2] Limeira-Soares P. H., Lira R. P. C., Arieta C. E. L., Kara-José N. (2007). Demand incidence of retinal detachment in Brazil. *Eye*.

[3] Sheldrick J. H., Vernon S. A., Wilson A., Read S. J. (1992). Demand incidence and episode rates of ophthalmic disease in a defined urban population. *British Medical Journal*.

[4] Rehman Siddiqui M. A., Abdelkader E., Hammam T., Murdoch J. R., Lois N. (2010). Socioeconomic status and delayed presentation in rhegmatogenous retinal detachment. *Acta Ophthalmologica*.

[5] Polkinghorne P. J., Craig J. P. (2004). Analysis of symptoms associated with rhegmatogenous retinal detachments. *Clinical & Experimental Ophthalmology*.

[6] Quinn S. M., Qureshi F., Charles S. J. (2004). Assessment of delays in presentation of patients with retinal detachment to a tertiary referral centre. *Ophthalmic and Physiological Optics*.

